# HSCT and CAR-T: a power couple—but who serves whom?

**DOI:** 10.3389/fimmu.2026.1898336

**Published:** 2026-07-21

**Authors:** Shuai Yao, Ran Zhang, Wenbin Mo, Zhenghua Liu, Heyang Zhang, Nan Su

**Affiliations:** Department of Hematology, The First Affiliated Hospital of China Medical University, Shenyang, Liaoning, China

**Keywords:** allogeneic transplantation, autologous transplantation, CAR-T, conditioning regimen, hematologic malignancies, hematopoietic stem cell transplantation

## Abstract

Hematopoietic stem cell transplantation (HSCT) and chimeric antigen receptor T-cell (CAR-T) therapy are two major therapeutic approaches for hematologic malignancies. HSCT can provide durable disease control through conditioning-induced tumor cytoreduction, immune reconstitution, and graft-versus-leukemia effects, whereas CAR-T therapy provides antigen-specific antitumor activity and has reshaped the treatment landscape for relapsed or refractory leukemia, lymphoma, and multiple myeloma (MM). Despite these advances, both therapeutic approaches continue to face multiple challenges. HSCT is limited by disease relapse and transplant-related complications, while CAR-T therapy is challenged by antigen escape, limited persistence, and treatment-related toxicities. Increasing clinical experience suggests that HSCT and CAR-T therapy should not be viewed as competing strategies, but rather as complementary therapeutic approaches whose value depends on disease characteristics, depth of response, immune recovery, and transplant feasibility. On the one hand, CAR-T therapy can reduce tumor burden before transplantation, be incorporated into modified conditioning regimens, or be used after allogeneic HSCT (allo-HSCT) as prophylactic, preemptive, or salvage cellular therapy. On the other hand, HSCT can consolidate CAR-T-induced remission, provide hematopoietic support in selected settings, and create a post-transplant immune environment that may facilitate subsequent CAR-T-cell therapy. In this review, we summarize the current evidence for HSCT–CAR-T integration in leukemia, lymphoma, and MM. We discuss major integration strategies, including HSCT after CAR-T therapy, CAR-T-assisted conditioning, autologous stem cell transplantation (ASCT)–CAR-T sequential strategies, and CAR-T therapy after allo-HSCT. We aim to help match appropriate patients to the most suitable HSCT–CAR-T strategies and thereby improve clinical decision-making.

## 1 Introduction

Hematologic malignancies encompass a broad range of disease entities with distinct patterns of progression, molecular genetic features, and sensitivity to therapy. Durable disease control depends not only on the initial treatment response, but also on the depth of remission and the feasibility of subsequent immune-based consolidation. Hematopoietic stem cell transplantation (HSCT) has long been a cornerstone therapy for patients with high-risk or relapsed/refractory (R/R) hematologic malignancies (1, 2). Autologous HSCT (ASCT) is widely used as consolidation therapy in multiple myeloma (MM) and some lymphomas, while allogeneic HSCT (allo-HSCT) provides donor-derived hematopoiesis and immune reconstitution in acute leukemia and other high-risk diseases, and mediates graft-versus-leukemia effects (1, 2). However, transplant-related complications, including graft-versus-host disease (GVHD), infections, and engraftment failure, limit the long-term benefits of HSCT. Moreover, relapse after transplantation remains a major treatment challenge (1, 3).

Chimeric antigen receptor T-cell (CAR-T) therapy offers a new treatment option for patients with disease refractory to conventional treatments through the targeted elimination of malignant cells expressing specific tumor-associated antigens (4–6). CD19-directed CAR-T therapies have shown promising efficacy in R/R B-cell acute lymphoblastic leukemia (B-ALL) and aggressive B-cell lymphomas, whereas B-cell maturation antigen (BCMA)-directed CAR-T therapies have improved outcomes in R/R MM. Other targets, including CD7, CD22, CD30, CD33, CD123, and C-type lectin-like molecule-1 (CLL-1), have expanded the potential application of CAR-T therapy to T-cell and myeloid malignancies (4–16). However, long-term remission after CAR-T therapy remains challenging for some patients, especially those with features associated with treatment failure or relapse (12, 17–22). Late toxicities associated with CAR-T therapy also require careful consideration (14, 16, 22–24).

**Figure 1:**
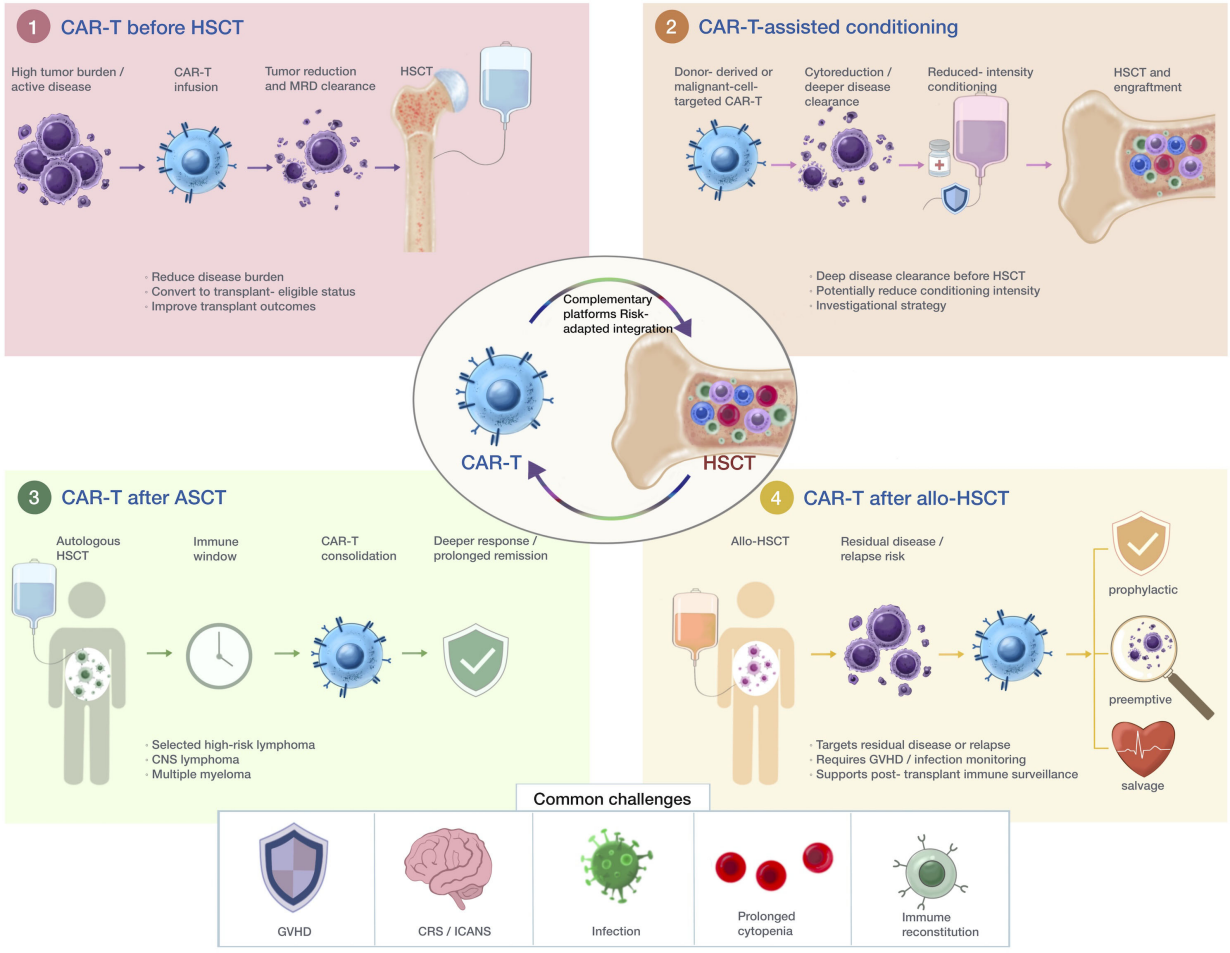
Risk-adapted integration of HSCT and CAR-T therapy in hematologic malignancies. The schematic summarizes four major strategies for integrating chimeric antigen receptor T-cell (CAR-T) therapy with hematopoietic stem cell transplantation (HSCT) (1). CAR-T therapy before HSCT may reduce tumor burden, promote measurable residual disease (MRD) clearance, convert active disease into a transplant-eligible state, and potentially improve post-transplant outcomes (2). CAR-T-assisted conditioning incorporates donor-derived or malignant-cell-targeted CAR-T cells into modified transplant conditioning, aiming to enhance cytoreduction, deepen disease clearance before HSCT, and potentially reduce conditioning intensity; however, this approach remains investigational (3). CAR-T therapy after autologous HSCT (ASCT) may exploit the post-transplant immune window to deepen response and prolong remission, particularly in selected high-risk lymphoma, central nervous system lymphoma, and multiple myeloma (4). CAR-T therapy after allogeneic HSCT (allo-HSCT) can be used as prophylactic, preemptive, or salvage cellular therapy to target residual disease or relapse, but requires close monitoring for graft-versus-host disease (GVHD), infection, and immune reconstitution. Across all integration strategies, common challenges include GVHD, cytokine release syndrome (CRS), immune effector cell-associated neurotoxicity syndrome (ICANS), infection, prolonged cytopenia, and delayed or incomplete immune reconstitution.

Together, these limitations have prompted renewed exploration of how CAR-T therapy can be integrated with HSCT to improve disease control in selected patients (17–19, 22–25). Rather than treating CAR-T therapy and HSCT as competing therapeutic options, the key question is how these approaches should be rationally sequenced or combined based on patient-specific risk profiles. This review summarizes the current evidence for HSCT–CAR-T integration in hematologic malignancies, focusing on four major strategies: CAR-T therapy before HSCT, incorporation of CAR-T cells into transplant conditioning or transplant-integrated platforms, ASCT followed by CAR-T-cell infusion, and CAR-T therapy after allo-HSCT as prophylactic, preemptive, or salvage cellular therapy. We also discuss safety, disease-specific patient selection, unresolved controversies, and future directions for individualized cellular therapy sequencing. **Figure 1** provides a conceptual overview of the major HSCT–CAR-T integration scenarios and key decision variables.

## 2 Literature search strategy and study selection

We conducted a narrative literature review to identify clinical studies evaluating the integration of HSCT and CAR-T therapy in hematologic malignancies. PubMed was searched from database inception to June 2026. Search terms included combinations of “CAR-T,” “chimeric antigen receptor T cell,” “hematopoietic stem cell transplantation,” “HSCT,” “allogeneic transplantation,” “allo-HSCT,” “autologous transplantation,” “ASCT,” “conditioning,” “donor-derived CAR-T,” “post-transplant relapse,” “minimal residual disease,” “measurable residual disease,” “acute lymphoblastic leukemia,” “acute myeloid leukemia,” “mixed-phenotype acute leukemia,” “lymphoma,” and “multiple myeloma.” Reference lists of relevant articles and available conference abstracts were also manually reviewed to identify additional eligible studies.

Studies were included if they reported clinical outcomes, safety data, or patient-selection considerations relevant to HSCT–CAR-T integration. Eligible evidence included clinical studies, case series, case reports, and selected conference abstracts when full-text data were unavailable for emerging strategies. Preclinical studies, non-hematologic malignancy studies, unrelated CAR-T or HSCT reports, duplicate publications, and articles with insufficient clinical information were excluded.

## 3 CAR-T therapy before HSCT

### 3.1 CAR-T before allo-HSCT in R/R B-ALL

For patients with R/R B-ALL, conventional salvage chemotherapy has limited efficacy, and durable remission remains difficult to achieve (26). CD19-directed CAR-T therapy can induce high remission rates and enable a substantial proportion of patients to achieve MRD-negative remission (7, 8, 27). However, whether CAR-T-induced remission should routinely be consolidated with allo-HSCT remains controversial.

Early studies raised the possibility that selected patients achieving MRD-negative complete remission (CR) after CAR-T therapy might not require immediate consolidative allo-HSCT. In a Memorial Sloan Kettering Cancer Center study of 53 patients with R/R B-ALL treated with 19-28z CAR-T cells, a subsequent analysis focused on 32 patients who achieved MRD-negative CR. No statistically significant difference in EFS or OS was observed between patients who underwent allo-HSCT and those who did not proceed to transplantation (n = 16 each; EFS: P = 0.64; OS: P = 0.89) (17).

Similar observations were reported in a Chinese non-randomized pragmatic trial of 58 patients with R/R B-ALL treated with CD19 CAR-T therapy. Among 51 patients who achieved CR or complete remission with incomplete hematologic recovery (CRi) within 1 month after infusion, 47 achieved MRD-negative CR. Outcomes were compared between 21 MRD-negative patients who underwent allo-HSCT at a median of 44 days after CAR-T therapy and 26 MRD-negative patients who did not receive transplantation, with no significant difference in OS between the two groups (P = 0.099) (18). Importantly, this study suggested that the benefit of allo-HSCT may depend on pre-infusion disease burden and adverse prognostic markers. Among patients with low disease burden before CAR-T infusion, defined as flow cytometry-assessed bone marrow MRD <5%, and without adverse markers, including complex karyotype, BCR-ABL1, MLL-AF4, TP53, or E2A-PBX1, consolidation allo-HSCT did not significantly improve RFS or EFS compared with no transplantation (n = 6 vs. 4; both P = 0.2207). In contrast, among patients with high pre-infusion bone marrow tumor burden, defined as MRD ≥5%, consolidation allo-HSCT significantly improved RFS and EFS regardless of adverse prognostic markers (n = 9 vs. 18; EFS: P = 0.032; RFS: P = 0.0031) (18). Overall, these early findings suggest that routine consolidative allo-HSCT may not be necessary for all patients who achieve MRD-negative CR after CAR-T therapy (17, 18).

However, subsequent larger retrospective studies suggested that patients achieving CR or MRD-negative remission after CAR-T therapy may still benefit from consolidative allo-HSCT to improve durable remission and survival outcomes. In a single-center analysis of 254 pediatric and adult patients with R/R B-ALL treated with CD19 CAR-T cells, 211 patients achieved CR and had available long-term follow-up. Among them, consolidative allo-HSCT was associated with superior survival compared with CAR-T therapy alone (n = 184 vs. 27), with higher 2-year OS (68.1% vs. 28.3%, P < 0.0001), and 2-year LFS (60.4% vs. 27.8%, P < 0.0001). The overall relapse rate during follow-up was also lower in the allo-HSCT group than in the CAR-T-alone group (17.4% vs. 40.7%, P = 0.009) (19). Importantly, subgroup analysis suggested that this benefit of consolidative allo-HSCT was not limited to patients with high pre-CAR-T tumor burden. Among 96 patients with pre-CAR-T bone marrow blasts ≤5%, those who proceeded to allo-HSCT (n = 81) had better 2-year OS (69.1% vs. 40.0%, P = 0.005) and LFS (65.7% vs. 46.7%, P = 0.014) than those who did not undergo transplantation (n = 15) (19).

This study also highlighted the potential value of consolidative allo-HSCT in genetically high-risk B-ALL. Among 23 patients with TP53 mutations, allo-HSCT after CAR-T therapy was associated with markedly improved 1-year OS (69.5% vs. 16.7%, P = 0.002) and LFS (58.8% vs. 16.7%, P = 0.004) compared with CAR-T therapy alone (19). Fusion genotype also appeared to influence the durability of CAR-T-induced remission and the expected benefit of allo-HSCT. Patients with E2A-PBX1 or E2A-HLF achieved high initial CR rates after CAR-T therapy but had poorer long-term disease control than patients with other fusion genes, including BCR-ABL1, MLL-AF4, and ETV6-RUNX1. In patients with E2A-PBX1, relapse remained frequent despite subsequent allo-HSCT, suggesting that transplantation alone may not fully overcome this high-risk biology. In the very small E2A-HLF subgroup, durable remission was observed only in patients who underwent allo-HSCT, whereas those who did not receive transplantation relapsed (19).

Other molecular abnormalities have also been examined in some small-scale CAR-T-to-HSCT studies. In a retrospective multicenter analysis by Lamble et al., KMT2A rearrangement was the only independent predictor of lineage switch relapse after CD19 CAR-T therapy (HR = 32.35; P < 0.0001). Seven KMT2A-rearranged patients underwent consolidative HSCT in remission at a median of 100 days after CAR-T therapy, and no post-HSCT lineage switch events were observed; by contrast, all seven KMT2A-rearranged patients who did not receive HSCT developed lineage switch relapse (21). Although limited by small numbers, these findings suggest that allo-HSCT may be particularly important after CAR-T-induced remission in patients at high risk of lineage switch or antigen-negative relapse. CAR-T-to-HSCT sequencing has also shown encouraging outcomes in Ph-like ALL. In a retrospective study of 17 patients with Ph-like B-ALL treated with autologous CD19 CAR-T or tandem CD19/CD22 CAR-T therapy followed by allo-HSCT, 16 achieved CR, including 11 MRD-negative and 5 MRD-positive remissions. The estimated 3-year OS exceeded 80% in both the JAK–STAT-activated and ABL1-class subgroups, with outcomes broadly comparable to Ph-positive and B-ALL-other cohorts treated using the same CAR-T-to-HSCT strategy (20). Together, these limited data suggest that molecular subtype may inform post-CAR-T transplant decisions, although larger subgroup-specific studies are needed.

In addition, retrospective transplant cohorts further suggest that allo-HSCT after CAR-T-induced CR can achieve post-transplant outcomes comparable to those after chemotherapy-induced CR, even when CAR-T-bridged patients have a more advanced remission history or adverse cytogenetic features before transplantation. In a cohort of 105 B-ALL patients undergoing allo-HSCT after CR induced by either CAR-T therapy (n = 27) or chemotherapy (n = 78), the CAR-T–bridged group had a higher proportion of patients transplanted in ≥CR2 (78% vs. 37%, P < 0.01) and a higher prevalence of complex cytogenetics (44% vs. 6%, P < 0.001). Nevertheless, 4-year transplant outcomes did not differ significantly between the CAR-T and chemotherapy groups, including CIR (11.1% vs. 12.8%, P = 0.84), LFS (70.2% vs. 64.1%, P = 0.63), and OS (70.2% vs. 65.4%, P = 0.681) (23). Similarly, in a retrospective study of 168 B-ALL patients undergoing haploidentical HSCT (haplo-HSCT) after MRD-negative CR induced by either CAR-T therapy (n = 28) or chemotherapy (n = 140), overall transplant outcomes were comparable between the CAR-T– and chemotherapy-bridged groups. In the MRD-negative ≥CR2 subgroup, however, CAR-T-bridged patients had better outcomes than chemotherapy-bridged patients, with higher 2-year OS (87.9% vs. 37.8%) and LFS (72.0% vs. 41.7%) (25).

In summary, these studies suggest that, in selected patients with R/R B-ALL, CAR-T therapy can induce a transplantable remission and serve as an effective bridge to allo-HSCT. Conversely, for patients who achieve CR or MRD-negative remission but remain at high risk of relapse because of adverse disease biology, consolidative allo-HSCT may extend the therapeutic benefit of CAR-T therapy.

### 3.2 CD7 CAR-T before allo-HSCT in R/R T-ALL/LBL

Beyond B-ALL, CD7-directed CAR-T therapy has been investigated as a bridge to allo-HSCT in R/R T-ALL/LBL. In a retrospective cohort of 90 transplant patients, Li et al. reported that chemotherapy-resistant patients receiving pre-transplant CD7 CAR-T therapy achieved 2-year OS and DFS rates of 54.4% and 51.0%, respectively (16). Among chemotherapy-resistant patients, the CD7 CAR-T–bridged group also had a lower 2-year cumulative incidence of relapse than patients undergoing salvage transplantation in non-remission without CAR-T bridging (31.67% vs. 70.5%) (16). These findings suggest that CD7 CAR-T therapy may help selected chemotherapy-resistant patients achieve better disease control before allo-HSCT.

This CAR-T-to-HSCT sequence was further evaluated in patients who achieved CR after CD7 CAR-T therapy. In a retrospective study by Cao et al., 34 patients with R/R T-ALL/LBL who achieved CR after autologous CD7 CAR-T therapy and subsequently underwent allo-HSCT were compared with 124 T-ALL/LBL patients who underwent allo-HSCT after chemotherapy-induced CR. The CD7 CAR-T and chemotherapy groups had comparable 2-year OS (61.9% vs. 67.6%, P = 0.210), LFS (62.3% vs. 62.0%, P = 0.548), NRM (32.0% vs. 25.3%, P = 0.288), and relapse incidence (8.8% vs. 15.8%, P = 0.557), supporting the feasibility of CD7 CAR-T-induced CR as a bridge to subsequent allo-HSCT in R/R T-ALL/LBL (24).

Although clinical evidence remains limited, available data suggest that CD7-directed CAR-T therapy may offer chemotherapy-resistant R/R T-ALL/LBL patients an opportunity to achieve disease control before allo-HSCT and thereby improve the chance of longer survival.

### 3.3 CAR-T before HSCT in R/R AML and MPAL

Unlike in lymphoid hematologic malignancies, CAR-T therapy has made only limited progress in AML, and available evidence is largely derived from small studies, mainly because most target antigens are not leukemia-specific. Commonly explored targets, including CD33, CD123, and CLL-1 (CLEC12A), are also expressed to varying degrees on normal hematopoietic progenitor or myeloid compartments. As a result, myeloid antigen-directed CAR-T therapy can cause profound and prolonged myelosuppression, making post-CAR-T hematopoietic support or transplant rescue a practical consideration (10, 11, 13–15).

In a first-in-human phase I study, 10 adults with R/R AML with a median CLL-1 positivity of 85.2% received CLL-1 CAR-T therapy, and 7 achieved CR/CRi, including 4 MRD-negative and 3 MRD-positive remissions. However, all patients developed severe pancytopenia, and 2 died from severe infection secondary to persistent agranulocytosis despite achieving CRi. Six patients underwent haplo-HSCT at a median of 20 days after CAR-T infusion (range, 18–34 days) to mitigate prolonged myelosuppression, followed by hematopoietic recovery, and 4 remained in CR at last follow-up. Among patients who did not undergo HSCT, 2 died from infection, 1 died from rapid disease progression, and 1 maintained durable CR without further therapy (14).

A minority of AML cases aberrantly express lymphoid-associated antigens such as CD7 or CD19, which may allow CAR-T targeting with less direct injury to normal hematopoietic stem cells than myeloid-lineage antigens such as CD33 or CD123. However, AML remains biologically heterogeneous and prone to antigen downregulation, antigen loss, and phenotypic switching, which may limit CAR-T durability (28, 29).

In a phase I trial of CD7-targeted CAR-T therapy, 10 patients with CD7-positive R/R AML were treated, and 7 achieved CR/CRi. Three responders proceeded to consolidative second allo-HSCT after CAR-T therapy, all of whom had relapsed after prior allo-HSCT. In contrast, among the 4 responders who did not receive consolidative allo-HSCT, 3 relapsed within 90 days and 1 died of pulmonary infection on day 48. CD7 loss was documented in all nonresponders and relapsed cases (12).

A similar pattern was observed in CD19-positive t (8, 21) AML. In a single-center prospective phase II trial, 10 patients with relapsed CD19-positive t (8, 21) AML achieved CR after CD19 CAR-T therapy, including 6 molecular MRD-negative remissions. However, relapse remained common, with 6 patients relapsing at a median of 3.4 months, including 5 CD19-negative relapses and 1 CD19-positive relapse. Three patients underwent allo-HSCT as post-CAR-T consolidation, but outcomes were mixed, including one ongoing remission and two deaths from post-transplant GVHD or relapse (9).

These challenges may be further amplified in MPAL. Evidence supporting CAR-T bridging to allo-HSCT in MPAL remains limited to very small case series. In the report by Kong et al., 5 patients with Ph-positive MPAL received CD19 or bispecific CD19/CD22 CAR-T therapy. One patient died from grade 5 cytokine release syndrome (CRS), whereas the other 4 patients showed marked reduction or clearance of BCR-ABL transcripts after CAR-T therapy and subsequently underwent haplo-HSCT within 3 months. All 4 transplanted patients maintained molecular CR at a median follow-up of 21.4 months (30). These findings suggest that, in selected patients with Ph-positive MPAL, CAR-T therapy may induce cytoreduction that can subsequently be consolidated by allo-HSCT. However, the evidence remains anecdotal, and the role of CAR-T-to-HSCT sequencing with other targets, such as CD7, CD20, and CD22, requires further investigation.

Overall, in AML and MPAL, CAR-T therapy is more likely to function as remission-inducing therapy than as definitive stand-alone treatment. In this context, allo-HSCT may help maintain CAR-T-induced remissions that might otherwise be short-lived because of antigen loss, phenotypic transformation, or early relapse, and may provide hematopoietic rescue when myeloid antigen-directed CAR-T therapy causes on-target hematopoietic toxicity.

### 3.4 Selective post-CAR-T transplant strategies in R/R MM and NHL

Outside acute leukemia, CAR-T and HSCT have also been combined or sequenced in MM and NHL, but the evidence is also limited (31–34).

In MM, the available evidence mainly concerns ASCT as selective consolidation after BCMA-directed CAR-T therapy rather than routine allo-HSCT after CAR-T-induced remission. In a retrospective cohort of 39 patients with R/R MM treated with BCMA-targeted CAR-T therapy, 13 underwent consolidative ASCT at a median of 92 days after CAR-T infusion, whereas 26 received maintenance therapy. Consolidative ASCT was associated with improved OS and PFS in multivariable analyses, suggesting that ASCT may be a feasible post-CAR-T consolidation option in selected patients, although this strategy remains non-routine and requires prospective validation (34).

In NHL, allo-HSCT after CAR-T therapy has been investigated mainly as salvage treatment after CAR-T failure rather than as routine consolidation after CAR-T-induced complete remission. In a multicenter retrospective study of 88 patients with R/R large B-cell lymphoma who underwent allo-HSCT after anti-CD19 CAR-T failure, the 1-year OS, PFS, and GVHD-free relapse-free survival (GRFS) were 59%, 45%, and 39%, respectively, while the 1-year NRM and progression/relapse rates were 22% and 33% (31). Complete remission at transplantation and fewer than two intervening lines of therapy between CAR-T and allo-HSCT were associated with better outcomes (31, 32).

Taken together, these disease-specific data suggest that the relationship between HSCT and CAR-T therapy outside acute leukemia is heterogeneous. Representative clinical studies informing transplantation strategies after CAR-T therapy across these disease settings are summarized in **Table 1**.

**Table 1:** Key clinical studies informing transplantation strategies after CAR-T therapy across hematologic malignancies.

Study	Disease	CAR-T target	Design and transplant strategy	Key outcomes	Clinical implication
Park et al., 2018 (17)	R/R B-ALL	CD19, 19-28z	Long-term adult follow-up; allo-HSCT vs observation after MRD-negative CR	No significant EFS or OS benefit from subsequent allo-HSCT among MRD-negative responders	Suggests that selected MRD-negative responders may not require routine immediate allo-HSCT; limited by small subgroup size and non-randomized design
Jiang et al., 2019 (18)	R/R B-ALL	CD19	Non-randomized pragmatic trial; MRD-negative responders underwent allo-HSCT or observation	Allo-HSCT improved EFS/RFS, especially in patients with high pre-infusion tumor burden	Supports risk-adapted rather than universal allo-HSCT after CAR-T therapy
Zhang et al., 2022 (19)	R/R B-ALL	CD19	Retrospective cohort; consolidative allo-HSCT vs CAR-T alone after CR	Allo-HSCT improved 2-year OS/LFS and reduced relapse	Provides large retrospective support for consolidative allo-HSCT after CAR-T-induced CR, but selection bias remains important
Yang et al., 2025 (22)	R/R B-ALL	CD19, CD19/CD22, or CD22	Long-term follow-up of MRD-negative responders undergoing allo-HSCT within 6 months after CAR-T	4-year OS 68.9%, LFS 61.4%, CIR 28.0%, NRM 10.6%	Supports feasibility and durability of sequential CAR-T followed by allo-HSCT; lacks non-transplant comparator
Lamble et al., 2023 (21)	KMT2A-rearranged ALL	CD19	Multicenter retrospective analysis of lineage switch relapse after CAR-T	No lineage switch events among seven KMT2A-rearranged patients receiving consolidative HSCT	Suggests allo-HSCT may mitigate lineage-switch risk in selected KMT2A-rearranged ALL
Dai et al., 2023 (20)	Ph-like B-ALL	CD19 or CD19/CD22	Retrospective cohort; CAR-T followed by allo-HSCT	3-year OS and RFS were comparable to Ph-positive and other B-ALL cohorts	Suggests CAR-T-bridged allo-HSCT may partly offset adverse prognosis in Ph-like ALL
Zhao et al., 2021 (23)	B-ALL in CR before allo-HSCT	CD19	Comparative cohort; allo-HSCT after CAR-T-induced CR vs chemotherapy-induced CR	Similar 4-year CIR, NRM, LFS, and OS between groups.	Indicates that CAR-T-induced CR can be an effective pre-transplant disease-control platform
Yang et al., 2022 (25)	B-ALL undergoing haplo-HSCT	CD19	Comparative cohort; haplo-HSCT after MRD-negative CR induced by CAR-T or chemotherapy	CAR-T-bridged patients achieved outcomes comparable to chemotherapy-bridged CR1 patients	Supports the feasibility of haplo-HSCT after CAR-T-induced MRD-negative remission
Aldoss et al., 2024 (35)	R/R B-ALL	CD19	Retrospective adult cohort; first or second allo-HSCT after CAR-T-induced CR	2-year OS 57.3%, RFS 56.2%, NRM 20.4%	Suggests that first or second allo-HSCT after CAR-T may be feasible in selected adults
Li et al., 2025 (16)	R/R T-ALL/LBL	CD7	Retrospective cohort; CD7 CAR-T bridging before allo-HSCT vs salvage HSCT without CAR-T	CD7 CAR-T bridging improved 2-year OS/DFS and reduced CIR compared with salvage HSCT	Supports CD7 CAR-T as a bridge to allo-HSCT in chemotherapy-resistant T-ALL/LBL
Cao et al., 2024 (24)	R/R T-ALL/LBL in CR before allo-HSCT	CD7	Comparative safety cohort; CD7 CAR-T-bridged vs chemotherapy-bridged allo-HSCT	Similar GVHD, viral infection, TA-TMA, PTLD, and NRM	Supports safety feasibility of CD7 CAR-T-bridged allo-HSCT, but not a formal non-inferiority trial
Jin et al., 2022 (14)	R/R AML	CLL-1	Phase I study; haplo-HSCT after CLL-1 CAR-T in responders with cytopenia	7/10 achieved CR/CRi; all developed severe pancytopenia; HSCT enabled hematopoietic recovery	Highlights that in AML, HSCT may function as both consolidation and hematopoietic rescue
Lu et al., 2025 (12)	CD7-positive R/R AML	CD7, NS7CAR-T	Phase I trial; selected responders underwent second allo-HSCT	7/10 achieved CR/CRi; non-transplanted responders frequently relapsed early	Suggests CD7 CAR-T may create a transplant window, but durability is limited by antigen loss and early relapse
Kong et al., 2020 (30)	Ph-positive MPAL	CD19 or CD19/CD22	Small case series; haplo-HSCT within 3 months after CAR-T	Four transplanted patients maintained molecular CR at median 21.4 months	Suggests CAR-T may create a transplant window in Ph-positive MPAL; evidence remains preliminary
Zhou et al., 2024 (34)	R/R MM	BCMA	Retrospective cohort; ASCT consolidation after BCMA CAR-T vs maintenance	ASCT consolidation was associated with improved OS and PFS	Supports exploration of ASCT after BCMA CAR-T in selected MM patients; disease logic differs from B-ALL
Zurko et al., 2023 (31)	R/R LBCL after CAR-T failure	Prior CD19 CAR-T	Multicenter retrospective cohort; allo-HSCT after CAR-T failure	1-year OS 59%, PFS 45%, NRM 22%	Represents a post-CAR-T failure salvage setting rather than routine post-CAR-T consolidation

This table includes representative studies in which transplantation was used after CAR-T therapy as consolidation, hematopoietic rescue, or salvage after CAR-T failure. The clinical rationale differs across disease entities: in B-ALL and T-ALL/LBL, HSCT is mainly discussed as post-CAR-T consolidation; in AML, HSCT may also serve as hematopoietic rescue after myeloid antigen-directed CAR-T therapy; in MM, available evidence mainly concerns ASCT consolidation after BCMA CAR-T; whereas in LBCL, allo-HSCT has primarily been explored after CAR-T failure rather than as routine consolidation after CAR-T-induced remission.

aGVHD, acute graft-versus-host disease; ALL, acute lymphoblastic leukemia; AML, acute myeloid leukemia; ASCT, autologous hematopoietic stem cell transplantation; CAR-T, chimeric antigen receptor T-cell therapy; cGVHD, chronic graft-versus-host disease; CIR, cumulative incidence of relapse; CMV, cytomegalovirus; CR, complete remission; CRi, complete remission with incomplete hematologic recovery; DFS, disease-free survival; EFS, event-free survival; GRFS, GVHD-free relapse-free survival; GVHD, graft-versus-host disease; HSCT, hematopoietic stem cell transplantation; LBCL, large B-cell lymphoma; LFS, leukemia-free survival; MM, multiple myeloma; MPAL, mixed-phenotype acute leukemia; MRD, measurable residual disease; NRM, non-relapse mortality; OS, overall survival; PFS, progression-free survival; PTLD, post-transplant lymphoproliferative disorder; R/R, relapsed/refractory; RFS, relapse-free survival; TA-TMA, transplant-associated thrombotic microangiopathy; T-ALL/LBL, T-cell acute lymphoblastic leukemia/lymphoblastic lymphoma.

### 3.5 Safety of allo-HSCT after CAR-T therapy

The safety of allo-HSCT after CAR-T therapy is a critical consideration when deciding whether CAR-T-induced remission should be followed by transplantation. Because directly comparable safety data are mainly available in ALL, this section focuses on CAR-T-to-allo-HSCT safety in B-ALL and T-ALL/LBL.

In a retrospective cohort of 105 R/R B-ALL patients who achieved CR and proceeded to allo-HSCT after either CD19 CAR-T therapy (n = 27) or chemotherapy (n = 78), major transplant-related complications were comparable between the CAR-T–bridged and chemotherapy-bridged groups. The cumulative incidences of grade III–IV aGVHD (11.1% vs. 11.5%, P = 0.945), extensive cGVHD (11.1% vs. 11.9%, P = 0.964), CMV reactivation (52% vs. 50%, P = 0.93), TA-TMA (15% vs. 14%, P = 0.51), and NRM (1-year: 11.1% vs. 16.7%; 4-year: 18.7% vs. 23.1%, P = 0.64) were similar between groups. Prior CRS grade did not significantly affect the incidence or severity of subsequent aGVHD (23). Longer-term follow-up data provide additional safety context. In a 51-patient cohort of R/R B-ALL patients who achieved MRD-negative CR after CAR-T therapy and subsequently underwent allo-HSCT, the day-100 cumulative incidences of grade I–IV and grade II–IV aGVHD were 31.4% and 15.7%, respectively, and the 4-year cumulative incidence of cGVHD was 48.3%. The 4-year NRM was 10.6%, and no GVHD-related deaths were reported (22).

Comparable safety observations have also been reported in T-cell malignancies. In a retrospective study of 158 patients with T-ALL/LBL who underwent allo-HSCT in CR, Cao et al. compared 34 patients with R/R T-ALL/LBL who achieved CR after CD7 CAR-T therapy with 124 patients who achieved CR after chemotherapy. No significant differences in major transplant-related complications were observed between the two groups. The incidences of any-grade aGVHD (44.1% vs. 44.7%, P = 0.848), 1-year any-grade cGVHD (31.6% vs. 28.3%, P = 0.758), 2-year TA-TMA (14.7% vs. 13.8%, P = 0.520), and 2-year NRM (32.0% vs. 25.3%, P = 0.288) were comparable. Viral infections, including CMV, EBV, and HHV-6, and post-transplant lymphoproliferative disorder also did not differ significantly between groups (24). These findings were reinforced by a more recent 90-patient R/R T-ALL/LBL transplant cohort, in which the CD7 CAR-T–bridged group showed no significant delay in white blood cell or platelet engraftment and no significant increase in aGVHD, cGVHD, bacterial or fungal infection, viremia, viral disease, CMV infection, or EBV infection compared with chemotherapy-sensitive patients transplanted in CR or patients undergoing salvage transplantation in non-remission (16).

Overall, available retrospective data have not shown a consistent signal that prior CAR-T therapy increases subsequent transplant-related toxicity, including GVHD, infection, endothelial complications, engraftment delay, or NRM, compared with chemotherapy-bridged transplantation. However, current evidence remains largely retrospective and subject to selection bias. Therefore, CAR-T–bridged allo-HSCT should be interpreted as feasible in carefully selected patients rather than universally safe across all disease settings.

### 3.6 Who and when? patient selection and timing of HSCT after CAR-T therapy

The optimal use of HSCT after CAR-T therapy depends on two clinically linked questions: who should proceed to transplantation, and when transplantation should be performed. Current evidence does not support a uniform strategy in which all patients achieving CAR-T-induced remission proceed directly to HSCT. Conversely, CAR-T-induced CR, even when MRD-negative, should not by itself be considered sufficient to omit transplantation.

#### 3.6.1 Who should proceed to HSCT after CAR-T therapy?

Patient selection should be individualized according to disease type, response depth, relapse risk, CAR-T persistence, toxicity, and transplant feasibility. In R/R B-ALL, the strongest rationale for post-CAR-T allo-HSCT exists in patients with high relapse risk. Factors supporting consolidative allo-HSCT include high pre-infusion disease burden, persistent or recurrent MRD after CAR-T therapy, poor CAR-T expansion or limited persistence, early loss of B-cell aplasia, extramedullary disease, prior exposure to CD19-directed immunotherapy, and high-risk genetic features such as TP53 alterations, KMT2A rearrangement, E2A-PBX1, E2A-HLF, and Ph-like genetic features (19–21). By contrast, patients with low pre-infusion disease burden, rapid achievement of MRD-negative CR, sustained CAR-T persistence, and no adverse molecular features may be considered for close surveillance rather than immediate transplantation, provided that intensive MRD monitoring and rapid intervention at molecular recurrence are feasible (17, 18). A history of allo-HSCT does not necessarily rule out a second transplant after CAR-T-induced remission, although this approach should be limited to carefully selected, fit patients. In a retrospective analysis of 45 adults with R/R B-ALL who achieved CR after CD19 CAR-T therapy and proceeded to allo-HSCT without interim therapy, outcomes were not significantly different between first allo-HSCT recipients (n = 26) and second allo-HSCT recipients (n = 19), with 2-year OS, RFS, CIR, and NRM of 57.3%, 56.2%, 23.3%, and 20.4% in the overall cohort (35).

Transplant fitness must also be incorporated into the “who” decision. Relevant factors include age, performance status, comorbidities, prior transplantation, donor type, conditioning intensity, infection history, cytopenia duration, organ dysfunction, and availability of post-transplant maintenance or immunomodulatory strategies. In older patients or in those with uncontrolled infection, persistent organ dysfunction, or severe ongoing cytopenias, the risk of NRM may outweigh the potential benefit of relapse reduction. Therefore, CAR-T–bridged HSCT should not be regarded as an automatic sequence, but as a dynamic, disease-specific strategy requiring repeated reassessment from CAR-T infusion through early response evaluation and pre-transplant workup.

#### 3.6.2 When should HSCT be performed after CAR-T therapy?

The timing of HSCT after CAR-T therapy should not be determined by calendar time alone but should be guided by relapse kinetics, MRD evolution, CAR-T persistence, toxicity recovery, hematopoietic status, and donor readiness. In general, earlier transplantation may be appropriate when relapse risk is high, MRD persists or recurs, CAR-T persistence is limited, target antigen escape is suspected, or hematopoietic rescue is required. Conversely, transplantation may be deferred in patients with sustained MRD-negative remission, ongoing CAR-T functional persistence, and substantial transplant-related risk, provided that intensive surveillance and rapid intervention are feasible.

MRD surveillance is central to timing decisions. Persistent MRD after CAR-T therapy, rising MRD during follow-up, or molecular recurrence should prompt timely evaluation for allo-HSCT in medically eligible patients. Serial MRD testing, antigen expression assessment, B-cell aplasia monitoring for CD19-directed products, and disease-specific molecular tracking may help identify impending relapse before overt hematologic recurrence (36, 37).

Before transplantation, acute CAR-T-related inflammatory toxicities, including CRS and ICANS, should generally have resolved, active infections should be controlled, organ function should have stabilized, and donor availability should have been secured. Overall, prospective studies using standardized MRD assessment and risk-adapted transplant criteria are needed to determine which patients should proceed to early consolidative HSCT and which patients may safely defer or omit transplantation.

## 4 CAR-T cells in transplant-integrated platforms

The strategies discussed in this section represent emerging approaches for integrating CAR-T therapy directly into the transplant process. Unlike CAR-T therapy as a bridge to allo-HSCT in B-ALL, which is supported by relatively broader clinical experience, CAR-T-assisted conditioning, conditioning-free or CAR-T-enabled engraftment, and CD7 CAR-T followed by haplo-HSCT without conventional GVHD prophylaxis remain at an early stage of clinical investigation. Most available evidence comes from case reports, small cohorts, or proof-of-concept studies. To avoid overinterpreting early evidence, **Table 2** summarizes the clinical maturity and interpretive limitations of these transplant-integrated CAR-T strategies.

**Table 2:** Clinical maturity and interpretive limitations of investigational HSCT–CAR-T integration strategies.

Strategy	Evidence base	Clinical maturity	Key safety and implementation concerns	Interpretative caution
CAR-T-assisted conditioning	Case reports and small cohorts using donor-derived CAR-T cells as an immune cytoreductive component of modified conditioning or transplant-integrated platforms (38, 39, 49)	Experimental; early proof-of-concept	CAR-T-cell dose and timing, conditioning intensity, donor source, CRS and ICANS, GVHD, infection, donor chimerism, immune reconstitution, and NRM	Biologically innovative but not established as standard clinical practice. It should be viewed as a proof-of-concept approach for highly selected patients in experienced centers.
Conditioning-free or CAR-T-enabled stem-cell engraftment	Isolated case reports in which stem-cell infusion was performed after CAR-T-induced marrow aplasia, immune suppression, or donor-derived immune chimerism (40, 52, 53)	Exceptional rescue strategy; case-report level evidence	Persistent cytopenia or marrow aplasia, infection, donor chimerism stability, need for GVHD prophylaxis, GVHD, TA-TMA, organ dysfunction, relapse, and transplant-related mortality	Should currently be interpreted as individualized hematopoietic rescue, not as a standardized conditioning-free transplant platform.
CD7 CAR-T followed by haploidentical HSCT without conventional GVHD prophylaxis	Small proof-of-concept cohort, case-based experience, and early clinical updates in CD7-positive hematologic malignancies (41, 42, 55)	Highly experimental; disease-specific proof-of-concept	Severe cytopenia, infection and viral reactivation, delayed immune reconstitution, CD7-negative relapse, GVHD risk, need for stem-cell rescue, and long-term safety	Promising, but currently limited to selected CD7-positive malignancies and experienced centers; prospective validation is required.

Clinical maturity and interpretive caution reflect the authors’ qualitative interpretation of the current evidence base rather than a formal evidence-grading system.CAR-T, chimeric antigen receptor T-cell therapy; CRS, cytokine release syndrome; GVHD, graft-versus-host disease; HSCT, hematopoietic stem cell transplantation; ICANS, immune effector cell-associated neurotoxicity syndrome; NRM, non-relapse mortality; TA-TMA, transplant-associated thrombotic microangiopathy.

### 4.1 Donor-derived CAR-T as part of modified conditioning regimens

In exploratory studies, donor-derived CAR-T cells have been investigated as part of modified HSCT conditioning regimens (38–40). Unlike conventional bridge-to-transplant strategies, CAR-T-assisted conditioning incorporates these cells into the transplant procedure to reduce tumor burden before donor graft infusion and subsequent hematopoietic reconstitution (39–42). This rationale may be particularly relevant for patients considered for reduced-intensity conditioning (RIC) because of age, frailty, or poor performance status, especially when high tumor burden or chemotherapy-resistant disease raises concern that overly attenuated conditioning may provide insufficient disease control (43). Conditioning chemotherapy may also create a lymphodepleted environment that supports CAR-T expansion and persistence (44, 45). Because the CAR-T cells and hematopoietic graft can be derived from the same donor, this approach may help overcome limitations of autologous CAR-T manufacture in heavily pretreated patients with impaired lymphocyte fitness, high tumor burden, rapid disease progression, or an immunosuppressive disease microenvironment (42, 46–48).

Early proof-of-concept experience in R/R ALL illustrated the feasibility of integrating donor-derived CAR-T cells into a transplant conditioning platform. Zhang et al. reported a 12-year-old girl with R/R CD19-positive ALL who underwent haplo-HSCT using an RIC regimen that incorporated haploidentical donor-derived CD19 CAR-T cells before donor stem-cell infusion. The patient achieved hematopoietic engraftment, full donor chimerism, and MRD-negative CR without severe CRS or GVHD (39).

A single AML case further illustrated both the potential and the risk of this strategy in myeloid disease. Yao et al. reported a patient with chemotherapy-resistant FUS–ERG-positive AML who relapsed shortly after prior allo-HSCT and remained in non-remission despite multiple salvage therapies. Donor-derived CD123 CAR-T cells were incorporated into an RIC-based haplo-HSCT platform, followed by stem-cell infusion from the same donor. Bone marrow blasts decreased within 2 weeks after CAR-T-cell infusion, and the patient achieved CRi, full donor chimerism, and myeloid engraftment. Unfortunately, grade IV aGVHD, severe infection, and multiorgan failure subsequently led to transplant-related death (49).

Additional experience comes from a small cohort of 8 adults with R/R B-NHL who underwent allo-HSCT using a conditioning regimen incorporating donor-derived CD19 CAR-T cells. Before transplantation, 5 patients were in partial remission, 1 had stable disease, and 2 had progressive disease. After this combined platform, 6 of 8 patients achieved CR, with 6-month OS and PFS rates of 75.0% and 62.5%, respectively. Notably, patients entering transplantation in partial remission appeared to have better outcomes than those with stable or progressive disease, suggesting that pre-transplant disease control remains important even in conditioning-integrated platforms (38).

Because CAR-T-assisted conditioning is delivered within the transplant process, its evaluation should extend beyond antitumor response to include CAR-T expansion and persistence, donor chimerism, engraftment kinetics, CRS, ICANS, GVHD, infections, immune reconstitution, prolonged cytopenia, endothelial complications, and NRM (38–42, 49–51). Based on the limited available evidence, this strategy appears more plausible in patients with chemotherapy-resistant but partially controlled disease than in those with uncontrolled progression, active infection, severe organ dysfunction, or unresolved inflammatory toxicities. Collectively, donor-derived CAR-T cells can be integrated into modified transplant conditioning across lymphoid and myeloid malignancies. However, the evidence remains preliminary and is derived mainly from case reports and a small cohort, with substantial heterogeneity in disease type, CAR-T target, conditioning intensity, donor source, pre-transplant disease status, and toxicity profile. Because conditioning chemotherapy, CAR-T-mediated cytoreduction, donor engraftment, and graft-versus-tumor effects occur in close temporal proximity, their relative contributions remain difficult to separate. These observations should therefore be regarded as hypothesis-generating evidence requiring further validation before CAR-T-assisted conditioning can be considered a reproducible conditioning strategy (38–42, 49).

### 4.2 CAR-T as the conditioning regimen

CAR-T therapy has also been reported to be followed by donor stem-cell engraftment without conventional conditioning in rare clinical cases. This situation differs from both planned CAR-T-to-HSCT bridging and CAR-T-assisted conditioning. In these cases, stem-cell infusion was not incorporated into a transplant platform in advance but was used after CAR-T therapy had produced profound marrow aplasia, host immune suppression, or donor-derived immune chimerism. Thus, conditioning-free stem-cell infusion should be viewed as an individualized hematopoietic rescue strategy rather than a reproducible conditioning approach (40, 52, 53).

Several case reports illustrate this concept across different diseases. In R/R Ph+ B-ALL, persistent marrow hypoplasia after anti-CD19 CAR-T therapy was followed by infusion of HLA-matched sibling hematopoietic stem cells without additional conditioning approximately 1 month later. The patient achieved MRD negativity, molecular remission, and full donor chimerism, although grade III aGVHD was subsequently considered likely (40).

In refractory Burkitt lymphoma, unedited HLA-matched allogeneic CD20/CD22 CAR-T therapy induced CR but was followed by persistent pancytopenia and full donor T-cell chimerism. Peripheral blood stem cells from the same donor were infused without conditioning on day 55 after CAR-T therapy, with subsequent neutrophil and platelet engraftment. No evident aGVHD occurred, although mild skin and ocular cGVHD later developed (53).

In R/R MM, a second BCMA-directed CAR-T infusion induced CR but was followed by refractory grade 4 myelosuppression and severe infections. Haploidentical peripheral blood stem cells were therefore infused without conventional conditioning or GVHD prophylaxis as hematopoietic rescue. Although early neutrophil recovery and high donor chimerism were observed, the patient later developed TA-TMA, grade III aGVHD, severe fungal infection, and died on day +222 (52).

Taken together, these isolated reports suggest that, in exceptional circumstances, CAR-T therapy may create conditions permissive for donor-cell engraftment. These cases should not be interpreted as evidence that conventional conditioning can be routinely omitted. In addition, autologous stem-cell rescue may also support hematopoietic recovery, but its impact on durable disease control, PFS, OS, and NRM compared with allogeneic stem-cell infusion remains unclear and requires further study (40, 52–54).

### 4.3 Sequential CD7 CAR-T therapy followed by haplo-HSCT without GVHD prophylaxis

A more integrated sequential strategy has recently been explored for CD7-positive hematologic malignancies, in which CD7 CAR-T therapy is followed by haplo-HSCT without conventional myeloablative conditioning or pharmacologic GVHD prophylaxis. Distinct from both CAR-T-assisted conditioning and conditioning-free rescue engraftment, this planned sequence integrates CAR-T-mediated disease control with subsequent haplo-HSCT within a single transplant-integrated strategy (42).

In a proof-of-concept study, 10 patients with R/R CD7-positive leukemia or lymphoma received CD7-directed CAR-T therapy followed by haplo-HSCT. Nine patients received haploidentical donor-derived CD7 CAR-T cells, whereas 1 received an off-the-shelf universal CD7 CAR-T product. All 10 patients achieved CRi after CAR-T therapy but developed grade 4 pancytopenia, and subsequently underwent haplo-HSCT at a median of 19 days after CAR-T-cell infusion (42). After HSCT, 8 patients achieved full donor chimerism, 1 patient showed autologous hematopoiesis, and 1 patient died on day 13 from septic shock and encephalitis. Acute GVHD occurred in 4 patients, including 1 case attributed to CAR-T therapy and 3 cases of HSCT-associated grade II aGVHD. No grade III–IV aGVHD or cGVHD was reported. With a median follow-up of 15.1 months after CAR-T therapy, 6 patients remained in MRD-negative CR without further treatment, 2 patients experienced CD7-negative relapse, and 1 patient died of disease progression. The estimated 1-year OS and DFS rates were 68% and 54%, respectively (42).

The rationale for this strategy is based on the dual activity of CD7-directed CAR-T cells. CD7 CAR-T cells can eliminate CD7-positive malignant cells while also depleting CD7-positive T and NK-cell compartments, which may reduce alloreactive lymphocyte populations involved in GVHD (42, 47, 48). However, this same biology introduces important safety concerns. Grade 4 pancytopenia in all patients indicates profound hematopoietic toxicity and underscores the need for timely stem-cell support. In addition, depletion of normal T and NK-cell compartments raises concern for infection and viral reactivation, whereas CD7-negative relapse highlights antigen escape as a major barrier to durable disease control (42, 47, 48).

Overall, this proof-of-concept cohort supports the feasibility of this CD7 CAR-T–haplo-HSCT sequence in selected CD7-positive hematologic malignancies. In addition, an interim phase 1 update of sequential CD7 CAR-T therapy followed by haplo-HSCT without GVHD prophylaxis has been reported in abstract form (55). Nevertheless, this approach remains investigational, with unresolved questions regarding safety, durability of disease control, infection risk, antigen escape, and reproducibility across broader CD7-positive disease populations.

## 5 CAR-T therapy after HSCT

### 5.1 CAR-T after ASCT

The sequential use of CAR-T therapy after ASCT has emerged as an investigational strategy to improve disease control in selected high-risk lymphoid malignancies. Rather than viewing the two as mutually exclusive choices, post-ASCT CAR-T therapy aims to combine ASCT-mediated cytotoxic debulking with subsequent CAR-T-mediated antigen-specific immune control. Mechanistically, the high-dose chemotherapy component of ASCT may lower tumor burden, while post-transplant lymphopenia and homeostatic cytokine release may support CAR-T-cell expansion and persistence (56, 57). This chemotherapy exposure may also modulate the tumor microenvironment, although the clinical relevance of these effects remains to be defined (58–65).

The available evidence for post-ASCT CAR-T therapy differs by disease setting, spanning non-randomized clinical studies and retrospective comparisons in aggressive B-cell NHL; retrospective cohorts in R/R primary or secondary CNS lymphoma; and small clinical studies or conference updates in newly diagnosed high-risk MM.

#### 5.1.1 Post-ASCT CAR-T integration in high-risk aggressive B-cell NHL

This approach has been explored mainly in relapsed or refractory aggressive B-cell NHL, particularly TP53-altered B-NHL and refractory DLBCL (66–68).

In a clinical study of TP53-mutated R/R aggressive B-cell NHL, 28 patients received BEAM-conditioned ASCT followed by sequential CD19/CD22 CAR-T-cell cocktail therapy, with CAR-T-cell infusion administered between days +2 and +6 after autologous stem-cell infusion. Among them, 26 patients achieved an objective response, including 23 complete responses, and the estimated 24-month PFS and OS were 77.5% and 89.3%, respectively. Compared with CD19/CD22 CAR-T-cell therapy alone, the sequential ASCT–CAR-T approach was associated with improved response and survival outcomes and a lower incidence of severe CRS (59). Longer-term follow-up of a larger R/R B-NHL cohort with TP53 alterations also showed higher 5-year OS (70.2% vs. 40.0%) and PFS (64.9% vs. 35.4%) in the sequential ASCT–CAR-T group than in the CAR-T-alone group, with a 5-year NRM of 10.7% (64).

Comparative data in R/R diffuse large B-cell lymphoma (DLBCL) are also consistent with a potential benefit of ASCT–CAR-T integration. In an analysis of two single-arm trials including 124 R/R DLBCL patients who were not in remission after bridging therapy, CD19/CD22 CAR-T-cell cocktail therapy combined with ASCT achieved a higher best overall response rate (ORR) than CAR-T-cell cocktail therapy alone (93.22% vs. 78.46%, P = 0.023). Median PFS and OS were not reached in the ASCT–CAR-T group, compared with 5.68 and 27.61 months, respectively, in the CAR-T-alone group (65).

Together, these non-randomized data suggest that ASCT–CAR-T integration may be worth further investigation in selected high-risk aggressive B-cell lymphomas, although prospective validation remains needed.

#### 5.1.2 Post-ASCT CAR-T therapy in central nervous system lymphoma

CNS lymphoma represents another setting in which ASCT followed by CAR-T therapy has attracted attention. Historically, patients with primary or secondary CNS lymphoma were often excluded from CAR-T trials because of concerns regarding neurotoxicity. With accumulating clinical experience, CAR-T therapy has increasingly been explored in selected patients with CNS involvement (57, 69).

A retrospective study compared three treatment strategies in 56 patients with R/R primary or secondary CNS lymphoma: ASCT followed by CD19/CD22 CAR-T-cell cocktail therapy (n = 29), CD19/CD22 CAR-T-cell cocktail therapy alone (n = 10), and chemoimmunotherapy (n = 17). The ASCT–CAR-T group achieved numerically higher ORR and CR rates than the CAR-T-alone and chemoimmunotherapy groups (ORR: 82.75% vs. 60.00% vs. 58.83%; CR: 72.41% vs. 50.00% vs. 41.12%). With a median follow-up of 16.73 months, the median PFS and OS were not reached in the ASCT–CAR-T group, and the 2-year PFS was significantly higher than that of the CAR-T-alone and chemoimmunotherapy groups (65.52% vs. 30.00%, P = 0.0321; 65.52% vs. 23.53%, P = 0.0043) (61). Another retrospective cohort evaluated ASCT followed by CAR-T therapy in 38 patients with R/R primary or secondary CNS lymphoma and active CNS involvement before treatment. The best ORR was 78.9%, and the estimated 1-year OS and PFS rates were 72.8% and 57.4%, respectively, at a median follow-up of 37.5 months. Severe CRS and ICANS each occurred in 13.2% of patients. In the SCNSL subgroup, patients with CNS-only involvement had a higher 1-year PFS rate than those with concurrent CNS and systemic involvement (83.3% vs. 38.5%, P = 0.030) (60).

Taken together, these retrospective data suggest that CNS involvement alone should not be considered an absolute contraindication to CAR-T therapy. However, safety remains a central concern in CNS lymphoma. Available studies have not shown a clear increase in grade ≥3 CRS or ICANS with ASCT–CAR-T therapy compared with CAR-T therapy alone. Because patients with CNS involvement may be more vulnerable to neurotoxicity due to blood–brain barrier disruption, local inflammation, and vascular permeability changes, ASCT followed by CAR-T therapy in CNS lymphoma should be accompanied by close neurologic monitoring, early recognition and management of CRS and ICANS, and individualized risk assessment (70, 71).

#### 5.1.3 Early post-ASCT CAR-T infusion in high-risk newly diagnosed MM

In MM, the relationship between ASCT and CAR-T therapy is more complex. Available evidence includes both CAR-T therapy after ASCT and ASCT as consolidation after CAR-T therapy, and these two sequencing strategies should be clearly distinguished (56, 63, 72).

In newly diagnosed high-risk MM, an early post-ASCT infusion strategy using sequential CD19- and BCMA-directed CAR-T cells has shown encouraging response depth. In one study of 10 newly diagnosed high-risk patients, CAR-T cells were infused at a median of 10 days after ASCT. By day 100, 8 patients achieved stringent CR and 2 achieved very good partial response. With a median follow-up of 42 months, 70% of patients maintained MRD negativity for more than 2 years (63). A subsequent 5-year follow-up update reported in abstract form described 37 newly diagnosed high-risk MM patients treated with this post-ASCT anti-CD19 and anti-BCMA CAR-T strategy, with an ORR of 100%, MRD negativity of 94.6% at a sensitivity of 10^−5^, and 60-month PFS and OS rates of 59.2% and 85.3%, respectively (73).

These early data suggest that post-ASCT CAR-T infusion may be feasible and associated with deep responses in high-risk newly diagnosed MM, although longer-term peer-reviewed data are still needed.

#### 5.1.4 Patient selection and CAR-T fitness after ASCT

Patient selection is critical for ASCT–CAR-T integration. Disease status, number of prior therapies, tumor burden, organ function, stem-cell availability, CAR-T manufacturing feasibility, and expected toxicity should be incorporated into treatment planning. Although achieving at least partial remission before ASCT has traditionally been considered important for transplant benefit, early ASCT–CAR-T studies suggest that some selected patients with stable or even progressive disease before transplantation may still achieve meaningful responses after the combined strategy. However, these findings should not be taken to mean that uncontrolled disease is an ideal setting for ASCT–CAR-T integration. In R/R CNS lymphoma, patients with more than four prior lines of therapy had lower complete response rates after ASCT followed by CAR-T therapy than those with four or fewer prior lines, suggesting that earlier integration may be preferable in selected patients (60).

Prior ASCT may influence subsequent CAR-T biology, although available data remain inconsistent across diseases. In MM, ASCT performed within one year before BCMA CAR-T infusion has been associated with changes in CAR-T-cell composition, including a lower proportion of naive CAR-T cells, suggesting that recent high-dose therapy may affect T-cell fitness before CAR-T manufacture (74). Conversely, in DLBCL, prior ASCT did not appear to compromise the efficacy or safety of subsequent CD19 CAR-T therapy in a retrospective cohort (75). These discrepant findings indicate that the impact of prior ASCT is likely disease- and context-dependent, influenced by timing, baseline T-cell fitness, and intervening therapies.

### 5.2 CAR-T after allo-HSCT

Relapse after allo-HSCT remains a major cause of treatment failure and is associated with poor prognosis. Conventional salvage strategies, including reduction of immunosuppression, chemotherapy, donor lymphocyte infusion (DLI), and second allo-HSCT, are often limited by chemoresistance, poor performance status, active infection, organ dysfunction, incomplete immune reconstitution, and risks of GVHD and NRM (76, 77).

In this setting, CAR-T therapy has emerged as a salvage option for target-positive relapse after allo-HSCT by providing antigen-specific antitumor activity beyond conventional graft-versus-leukemia effects (78). CAR-T cells may be manufactured from the original stem-cell donor, recipient-collected T cells, or unrelated allogeneic products. In patients with complete donor chimerism, recipient-collected T cells may already be largely donor-derived, making chimerism status an important consideration in source selection. Other factors include donor availability, prior GVHD, disease kinetics, manufacturing feasibility, and treatment urgency (78).

The largest body of clinical experience comes from patients with B-ALL who relapse after allo-HSCT. In available studies, reported CR rates with donor-derived CD19 CAR-T therapy have ranged from approximately 79%–89.5% in this setting (79, 80). Long-term follow-up provides additional evidence that remission can be durable in a subset of patients. In a cohort of 32 patients with B-ALL relapsing after allo-HSCT who received donor-derived CD19 CAR-T cells, the 2-year OS and EFS were 56.25% and 50.0%, respectively, while the 5-year OS and EFS were 53.13% and 46.88%, respectively, with no new long-term adverse events reported (81). Prospective phase I data with donor-derived anti-CD19 CAR-T cells also showed *in vivo* CAR-T-cell expansion and MRD-negative CR/CRi in all evaluable patients at day 28. At a median follow-up of 475 days, 7 patients remained in CR/CRi, whereas 2 developed CD19-negative relapse; the 1-year PFS and OS were 77.8% and 85.7%, respectively. However, toxicity remained clinically relevant, with CRS and hematologic adverse events occurring in all patients and aGVHD occurring in 3 patients, including 1 grade IV case (82).

Retrospective comparisons suggest that donor-derived CAR-T therapy may provide more effective disease control than DLI-based salvage in selected patients with post-transplant B-ALL relapse. In one study comparing donor-derived CD19 CAR-T therapy with DLI, the CAR-T group had a higher MRD-negative CR rate and longer median OS, whereas severe GVHD was more frequent after DLI (83). Another retrospective study comparing donor-derived CD19 CAR-T therapy with chemotherapy plus DLI showed higher CR and MRD-negative CR rates in the CAR-T group than in the chemo-DLI group (77.3% and 61.5% vs. 38.1% and 23.8%). The 2-year LFS and OS rates were also higher with CAR-T therapy than with chemo-DLI (50.0% and 54.5% vs. 4.8% and 9.5%). CRS remained common after CAR-T therapy, occurring in 86.4% of patients, including grade III CRS in 27.3%, whereas grade II–IV aGVHD was more frequent in the chemo-DLI group (84).

Despite high initial response rates, durable remission after donor-derived CD19 CAR-T therapy remains challenging. One study reported a 1-year CIR of approximately 41% among post-allo-HSCT relapsed patients who achieved CR after donor-derived CD19 CAR-T therapy but did not receive subsequent consolidation (79). Sequential antigen targeting may help address antigen-negative relapse and improve remission durability. In patients with B-ALL relapsing after allo-HSCT, 30 patients received CD19 CAR-T therapy and 24 subsequently received CD22 CAR-T therapy. Among those treated with both CD19- and CD22-directed CAR-T cells, the 5-year EFS and OS were 50% and 75%, respectively (80). These data support the feasibility of sequential antigen targeting, but its role as post-remission consolidation remains undefined. Second allo-HSCT after CAR-T-induced remission may be considered in highly selected patients with MRD-negative CR, acceptable transplant fitness, good organ function, controlled infection, and available donor options. However, given its substantial toxicity, second allo-HSCT should not be considered routine for all patients achieving CAR-T-induced remission (77, 85).

Donor-derived CAR-T therapy has also been explored in myeloid relapse after allo-HSCT. In a recent study of donor-derived CLL-1 CAR-T cells for AML relapsing after allo-HSCT, 14 patients were enrolled and 12 received infusion. Among treated patients, 7 achieved MRD-negative CR and 2 achieved MRD-positive CR, corresponding to response rates of 58.33% and 16.67%, respectively (10). These findings suggest that donor-derived CLL-1 CAR-T cells may have antileukemic activity in post-transplant AML relapse, but toxicity was substantial, with CRS in all treated patients, grade ≥3 CRS in 5 patients, and CAR-T-cell-related encephalopathy syndrome (CRES) in 3 patients, including 1 grade IV event. Therefore, this strategy remains investigational and should be considered only with careful assessment of antigen expression, disease burden, marrow reserve, infection status, and feasibility of hematopoietic rescue or consolidation (10, 78).

GVHD remains a major safety concern for donor-derived CAR-T therapy after allo-HSCT. Several studies have reported post-infusion GVHD, including severe aGVHD (86–88). Donor-derived CAR-T cells may retain alloreactive potential or amplify pre-existing alloimmune activation, particularly in patients with prior GVHD, recent DLI exposure, HLA disparity, haploidentical donor source, or marked inflammatory activation after CAR-T infusion (78, 89, 90). By donor source, haploidentical donor-derived CAR-T products may carry higher GVHD risk than HLA-matched donor-derived products (82, 87). These findings indicate that GVHD prevention, early recognition, and close post-infusion monitoring are central to the safe use of donor-derived CAR-T therapy after allo-HSCT.

Overall, CAR-T therapy for relapse after allo-HSCT is best established in B-ALL, whereas evidence in AML and other settings remains limited. Its broader use is constrained by antigen escape, limited CAR-T persistence, GVHD, infection, cytopenia, and unresolved questions regarding consolidation after CAR-T-induced remission. Future studies should define the optimal CAR-T-cell source, dose, timing, target selection, GVHD monitoring strategy, and the role of second allo-HSCT or maintenance therapy.

### 5.3 Prophylactic and preemptive CAR-T therapy after allo-HSCT

In addition to salvage CAR-T therapy for overt hematologic relapse after allo-HSCT, CAR-T cells have also been explored as prophylactic or preemptive post-transplant interventions. In this review, prophylactic CAR-T therapy refers to CAR-T-cell infusion in patients without detectable relapse, MRD positivity, or molecular recurrence but with high-risk post-transplant features, whereas preemptive CAR-T therapy refers to infusion triggered by MRD positivity or molecular recurrence before overt hematologic relapse. This distinction is important because CAR-T therapy delivered at frank relapse is usually administered at a higher disease burden and may have different efficacy, toxicity, and relapse patterns (91–94). Accordingly, earlier CAR-T intervention aims to eliminate residual malignant clones at a low disease-burden stage before clinical relapse becomes established (92, 94–96).

Available evidence is concentrated in high-risk B-ALL after allo-HSCT. In one study, 23 high-risk B-ALL patients received prophylactic donor-derived CD19 CAR-T-cell infusion at a median of 122 days after transplantation and were compared with 44 high-risk patients who did not receive post-transplant maintenance therapy. Prophylactic CAR-T-cell infusion was associated with a lower 2-year CIR than observation alone (5.6% vs. 28.8%) and superior 2-year PFS (84.0% vs. 57.3%), although the 2-year OS difference was not statistically significant (89.3% vs. 75.4%). Toxicity was acceptable but not negligible: grade 1/2 CRS occurred in 47.8% of patients, no grade III-IV CRS or ICANS was reported, and 3 patients developed aGVHD, including 2 grade II cases and 1 grade III case. Infection-related deaths occurred in 3 patients, although the 2-year NRM was comparable between the CAR-T and control groups (91).

Preemptive CAR-T therapy has also been evaluated in MRD-positive B-ALL after allo-HSCT. In a prospective study, 12 MRD-positive patients received donor-derived CD19 CAR-T-cell infusion, whereas 21 MRD-positive patients who received DLI were analyzed as historical controls. All 12 patients in the CAR-T group achieved MRD-negative remission, with faster MRD clearance than the DLI group (median, 14 vs. 43 days). The safety profile appeared favorable: 8 patients experienced grade I CRS and 4 experienced no CRS after CAR-T infusion. No aGVHD, severe infection, CMV infection, or EBV infection was observed. By contrast, grade II–III aGVHD occurred more frequently after DLI (93).

The optimal timing of prophylactic or preemptive CAR-T infusion after allo-HSCT remains uncertain. Early infusion may be limited by delayed hematopoietic recovery, active infection, organ dysfunction, ongoing GVHD, or intensive immunosuppression, whereas delayed infusion may permit residual disease expansion (97, 98). Timing should therefore be individualized according to MRD kinetics, relapse risk, hematopoietic recovery, GVHD status, immunosuppressive exposure, and donor-derived CAR-T-cell availability. Beyond timing, the MRD threshold for intervention remains undefined and may be influenced by assay sensitivity, target antigen expression, immune reconstitution, and post-transplant inflammation (91, 93, 94). Post-transplant immunosuppression remains another unresolved issue. Calcineurin inhibitors, corticosteroids, and other GVHD-directed therapies may impair CAR-T-cell expansion, persistence, and effector function, whereas reducing immunosuppression to enhance CAR-T activity may increase the risk of GVHD or inflammatory complications. This balance is particularly important for donor-derived CAR-T products, which may mediate both antigen-specific antitumor effects and alloimmune toxicity (92, 94–96).

Overall, prophylactic or preemptive CAR-T therapy after allo-HSCT should be viewed as a risk-adapted post-transplant immune intervention rather than conventional maintenance therapy. Prospective studies are needed to define how prophylactic and preemptive CAR-T therapy should be integrated into post-transplant relapse-prevention strategies.

## 6 Discussion

As CAR-T therapy is increasingly used in earlier treatment settings, HSCT and CAR-T therapy should no longer be considered simply as competing options. The more relevant question is which patients need transplantation after CAR-T therapy, which patients may avoid it, and in which settings the two approaches should be combined.

First, CAR-T therapy can be used before subsequent HSCT, but its function differs by disease. In acute lymphoblastic leukemia, CAR-T therapy can serve as a remission-inducing or disease-control bridge to allo-HSCT. This strategy is best established in R/R B-ALL, particularly in patients with persistent or recurrent MRD, adverse molecular features, high pre-infusion tumor burden, or limited expected CAR-T persistence (17–25). In R/R T-ALL/LBL, CD7-directed CAR-T therapy may provide a similar bridging function for chemotherapy-resistant patients who would otherwise undergo transplantation in non-remission (16, 24). In AML and MPAL, CAR-T-induced responses are often transient and may be limited by antigen heterogeneity, antigen loss, and marrow toxicity, making HSCT relevant for both remission consolidation and hematopoietic rescue (9, 12, 14, 30). Outside acute leukemia, post-CAR-T transplant strategies remain more selective, including ASCT consolidation after BCMA-directed CAR-T therapy in MM and allo-HSCT as salvage after CAR-T failure in NHL, rather than routine consolidation for all responders (31–34). In this context, CAR-T therapy may create a transplant opportunity for patients who are otherwise unlikely to proceed directly to HSCT, while subsequent transplantation may further consolidate CAR-T-induced remission.

Second, CAR-T cells may be integrated directly into the transplant process rather than used only before or after HSCT. This category includes donor-derived CAR-T-assisted conditioning, conditioning-free hematopoietic rescue after CAR-T-induced marrow aplasia, and planned CD7 CAR-T therapy followed by haploidentical HSCT without conventional GVHD prophylaxis, with early experience reported mainly in R/R ALL, AML, B-NHL, and CD7-positive T-cell malignancies (38–42, 49–55). Such strategies differ mechanistically, but they share the broader goal of using CAR-T-mediated cytoreduction or immune modulation to facilitate donor hematopoiesis. At present, however, they remain exploratory and should be considered only in highly selected patients, with careful attention to disease control, infection status, organ function, graft source, GVHD risk, immune reconstitution, and feasibility of hematopoietic recovery.

Third, ASCT–CAR-T integration represents a distinct sequential strategy in which high-dose chemotherapy and autologous stem-cell rescue are followed by early CAR-T-cell infusion. The rationale is not simply to combine two intensive therapies, but to use ASCT as a cytotoxic and immunologic platform that reduces tumor burden, restores hematopoiesis, and creates a lymphodepleted post-transplant environment that may support subsequent CAR-T-cell expansion and antigen-specific disease control. This model has been explored mainly in aggressive B-cell lymphoma, CNS lymphoma, and MM (26, 56, 59–65, 72–75). ASCT–CAR-T integration should be considered a selective intensification strategy for patients with adequate fitness, at least partial chemosensitivity, and high-risk features, rather than a routine sequence for all patients eligible for CAR-T therapy.

Fourth, after allo-HSCT, CAR-T therapy should be stratified according to relapse burden and intervention intent. For overt hematologic relapse, CAR-T therapy functions primarily as salvage treatment, with decisions guided by target antigen expression, disease kinetics, chimerism status, donor availability, prior GVHD, infection status, and hematopoietic reserve. For MRD positivity or molecular recurrence, preemptive CAR-T therapy aims to intensify antigen-specific graft-versus-leukemia activity before frank relapse develops. For high-risk patients without detectable relapse, prophylactic CAR-T therapy remains investigational and should be considered only in carefully selected settings (78–96). Across all three scenarios, the major practical challenge is balancing CAR-T-cell expansion and antitumor activity against GVHD risk, ongoing immunosuppression, infection susceptibility, and delayed immune reconstitution.

It is worth noting that MRD is particularly useful for guiding individualized decisions on whether and when HSCT or CAR-T therapy should be used. After CAR-T therapy, persistent or recurrent MRD, particularly in the context of high-risk genetics, high pre-infusion tumor burden, or declining CAR-T persistence, may support consideration of HSCT consolidation. Conversely, sustained MRD negativity with durable CAR-T persistence may justify close surveillance rather than immediate transplantation in patients with high transplant-related risk. After allo-HSCT, MRD positivity or molecular recurrence may shift the strategy toward preemptive CAR-T therapy, whereas prophylactic CAR-T therapy in MRD-negative patients should remain restricted to high-risk settings and prospective evaluation. In this way, MRD can help determine whether HSCT and CAR-T therapy should be sequenced, combined, delayed, or avoided (36, 37, 94).

Despite encouraging signals, the current evidence base remains limited. Many available studies are retrospective, single-center, or based on small cohorts, and newer approaches such as CAR-T-assisted conditioning, conditioning-free donor-cell infusion, ASCT–CAR-T integration, and prophylactic or preemptive post-transplant CAR-T therapy are still supported mainly by case reports, early-phase studies, or non-randomized data. Selection bias is a major concern. Patients who are able to proceed to HSCT after CAR-T therapy, receive CAR-T therapy after HSCT, or undergo sequential ASCT–CAR-T integration are often fitter, more responsive to prior therapy, or more clinically stable than those who do not. Better outcomes in these groups may therefore partly reflect patient selection rather than the effect of the integrated strategy itself. Cross-study comparisons are also difficult because studies differ in disease biology, disease burden, MRD assessment, CAR-T product design, transplant platform, follow-up duration, and post-treatment management. Overall, the observed associations between HSCT–CAR-T integration and improved outcomes should be interpreted cautiously and should not be generalized as a universal strategy across disease settings.

Next-generation cellular therapies may further change how HSCT and CAR-T therapy are integrated. Dual-target or sequential CAR-T strategies may reduce antigen escape, donor-derived CAR-T cells may be better suited to post-transplant relapse or transplant-integrated platforms, and off-the-shelf or gene-edited products may shorten manufacturing time and overcome poor autologous T-cell fitness (42, 47, 55, 79–82). However, these advances should not be assumed to eliminate the need for HSCT; rather, they may redefine which patients require transplantation, which patients can safely avoid it, and which settings may benefit from donor immune reconstitution.

Future studies should define which patients are most likely to benefit from HSCT–CAR-T integration in each disease setting. Important factors include treatment line, MRD kinetics, antigen expression, CAR-T persistence, T-cell fitness, donor type, prior transplantation, conditioning intensity, immunosuppressive exposure, and how treatment response should guide subsequent transplant decisions. Endpoints should extend beyond CR and early MRD negativity to assess whether remission is durable, how relapse occurs, how immune recovery proceeds, and what long-term toxicities or treatment burdens patients experience (50, 51, 94–96, 99). Ultimately, the clinical value of HSCT–CAR-T integration will depend on its ability to maximize durable immune-mediated disease control while avoiding unnecessary treatment-related toxicity.
